# Antimicrobial activity of *Janthinobacterium, Pseudomonas* , and *Pseudoclavibacter* bacterial soil isolates

**DOI:** 10.17912/micropub.biology.001644

**Published:** 2026-04-08

**Authors:** Kelena Snipes, Logan Angelillo, Julia Fugate, Hannah-May Winton, Elias Taylor-Cornejo

**Affiliations:** 1 Department of Biology, Randolph–Macon College, Ashland, Virginia, United States

## Abstract

Healthcare, and the world at large, are currently facing an antibiotic crisis caused by the increase in antibiotic resistant bacterial pathogens, combined with divestment in the drug discovery pipeline for new antibiotics. The Tiny Earth Project is a global, student-driven, effort aimed at discovering and isolating new sources of antibiotics from soil microbes that can be used to combat multi-drug resistant bacterial pathogens. This study characterizes the antimicrobial and biochemical properties of three unique bacterial soil isolates from the genera *Janthinobacterium* , *Pseudomonas* , and *Pseudoclavibacter* and their extracted secondary metabolites.

**Figure 1. Antibiotic activity and biochemical characterization of bacterial soil isolates:** **(A)** Soil isolate ID TEPI010-014. TEPI010 is a known antibiotic-producing soil bacterium *Lysobacter antibioticus* that is used as a positive control for all experiments. **(B)** Soil isolate genus identified by 16S ribosome subunit gene sequencing. **(C)** Antibiotic screen for each soil isolate patched on top of the Gram-positive tester strain *Bacillus subtilis* . **(D)** Antibiotic screen for each soil isolate patched on top of the Gram-negative tester strain *Escherichia coli* . **(E)** Disc diffusion assay testing the antimicrobial susceptibility of Gram-positive tester strain *Bacillus subtilis* to antibiotic extracts from each soil isolate using a disc diffusion assay. **(F)** Mean ZOI in millimeters (mm) for the Gram-positive disc diffusion assay. Mean ZOI is the average of two diameter measurements across the ZOI shown. The mean ZOI was not determined (n.d.) for weak growth inhibition because the ZOI could not be measured with reliability. **(G)** Disc diffusion assay testing the antimicrobial susceptibility of Gram-negative tester strain *Escherichia coli* to antibiotic extracts from each soil isolate using a disc diffusion assay. **(H)** Mean ZOI in millimeters (mm) for the Gram-negative antimicrobial susceptibility test. Mean ZOI is the average of two diameter measurements across the ZOI shown. **(I)** Gram stain of each soil isolate. All brightfield images were taken at 100X magnification. **(J)** Gram-negative selection on MacConkey agar indicated by patch growth, lactose fermentation indicated by either pink patches (lactose-fermenting positive) or pale patches surrounded by yellow color (lactose-fermenting negative). **(K)** Catalase activity indicated by the presence/absence of bubbles from each isolate spread on a glass slide and treated with hydrogen peroxide. **(L)** Hemolysis on blood agar indicating alpha, beta or gamma hemolysis activity.

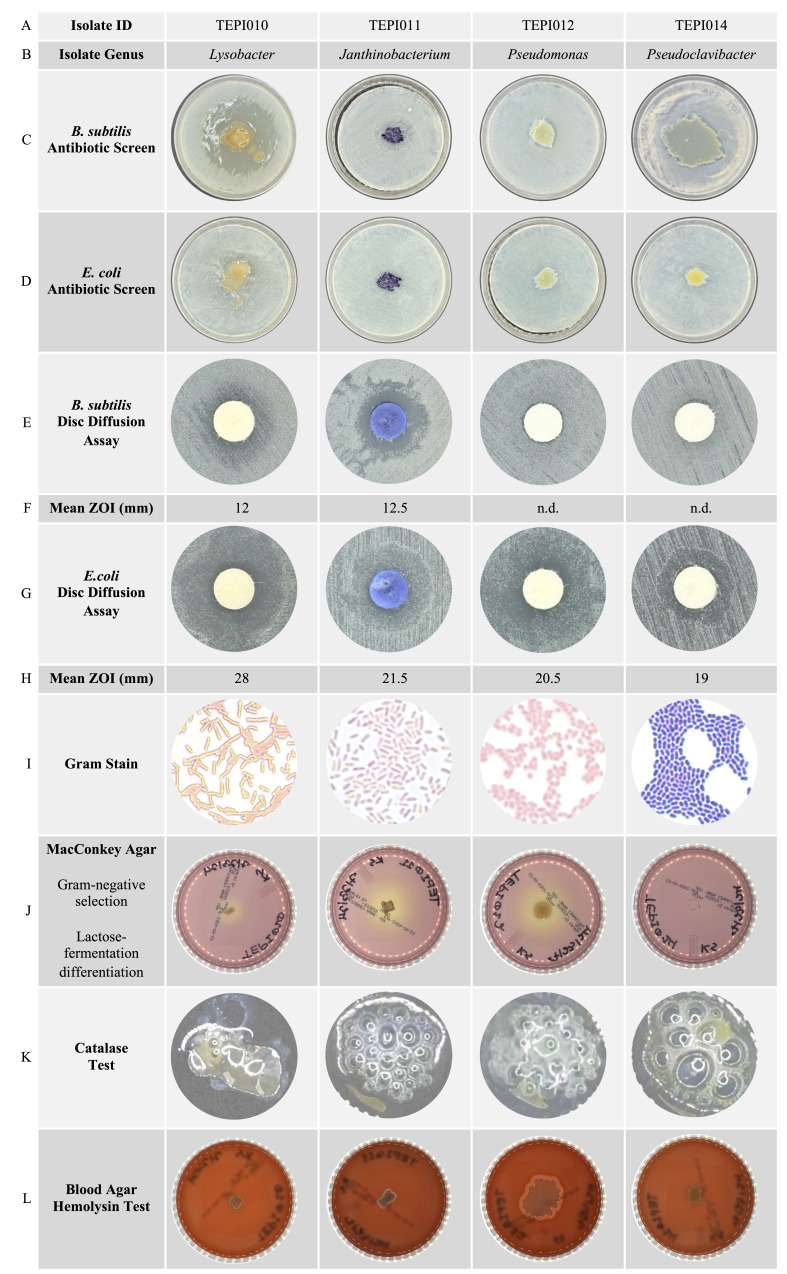



## Description

The rise of antimicrobial resistant (AMR) bacterial pathogens has placed a significant burden on the global healthcare system (Theuretzbacher et al. 2025). The growing number of multidrug resistant bacterial pathogens is outpacing the development of new antibiotics to treat infections by these pathogens (Martens and Demain 2017; Ventola 2015). Antibiotic discovery through the Tiny Earth Project represents a student-driven effort to combat this antibiotic crisis (Hurley et al. 2021). In this study, soil bacteria were isolated through the Tiny Earth Project at Randolph-Macon College in Ashland, VA by undergraduate microbiology students, who performed the initial sampling and culturing of bacterial soil isolates, as well as the preliminary antimicrobial screening and biochemical characterization of a soil isolate library.

Three soil isolates (TEPI011, TEPI012, and TEPI014) were selected for further validation and chemical extraction of antibiotics ( Figure 1A ). For all tests performed, the bacterial soil isolates were compared to a positive control strain, *Lysobacter antibioticus* ( *L. antibioticus* , TEPI010), *&nbsp;* a known antibiotic-producing bacterium (Christensen and Cook 1978). The genus of each soil isolate was determined using Oxford Nanopore Technology (ONT) Sequencing to obtain a full-length 16S rRNA gene sequence for each soil isolate (approximately 1,500 base pairs). We found that TEPI011 shares a 99.87% identity with *Janthinobacterium* , TEPI012 shares a 99.54% identify with *Pseudomonas* , and TEPI014 shares a 99.66% identify with *Pseudoclavibacter,* which meet the established criteria for genus-level identification from a 16S rRNA gene sequence ( Figure 1B ) (Janda and Abbott 2007). The antimicrobial activity of each soil isolate towards a Gram-negative tester strain, *Escherichia coli* *(E. coli)* , or a Gram-positive tester strain, *Bacillus subtilis* *(B. subtilis)* , was validated by first spreading the tester strain on the surface of a 10% TSA agar plate, then patching each soil isolate on top of the tester strain ( Figure 1C and 1D). The positive control, *L. antibioticus,* exhibited strong antimicrobial activity against Gram-positive bacteria and, to a lesser extent, Gram-negative bacteria, as previously described ( Figure 1C and 1D) (Christensen and Cook 1978; Kudryakova et al. 2023). Likewise, all of the soil isolates showed clear antimicrobial activity against *B. subtilis* and, to a lesser extent, antimicrobial activity against *E.coli* ( Figure 1C and 1D). Ethyl acetate chemical extractions of the organic secondary metabolites were performed for each isolate. The antimicrobial susceptibility of *B. subtilis* and *E.coli* to each extract was tested using a disc diffusion assay, which measures the mean zone of inhibition (ZOI) created around a paper disc containing each chemical extract ( Figure 1E- 1H). When the extracts were tested against the Gram-positive tester strain *B. subtilis,* TEPI011 (ZOI = 12.5 mm) showed the strongest antimicrobial activity, which was similar to that of the positive control extract from *L. antibioticus* (ZOI = 12 mm) and was substantially greater than the negative control (ZOI = 8 mm) ( Figure 1E- 1F and Extended Data). Extracts from the other soil isolates (TEPI012 and TEPI014) also created a visible ZOI against *B.* *subtilis,* but the exact boundary of the zones were too faint to measure reliably ( Figure 1E and 1F). All extracts showed antimicrobial activity against *E.coli* that was substantially greater than the negative control (ZOI = 11.5 mm): TEPI010 (ZOI = 28 mm), TEPI011 (ZOI = 21.5 mm), TEPI012 (ZOI = 20.5 mm), and TEPI014 (ZOI= 19 mm) ( Figure 1G- 1H and Extended Data).

Biochemical characterization for each soil isolate was performed using both selective and differential media, Gram staining, and catalase testing. TEPI011 and TEPI012 stained Gram-negative and grew on MacConkey agar, which selects for Gram-negative bacteria; on the other hand, TEPI014 stained Gram-positive and failed to grow on MacConkey agar ( Figure 1I and 1J). MacConkey agar also differentiates the ability to ferment lactose; TEPI011 and TEPI012 were shown to not be able to ferment lactose, as indicated by the yellow discoloration observed around the TEOI011 and TEPI012 isolates growing on MacConkey agar ( Figure 1J ). We tested for the presence of a catalase enzyme in each soil isolate, which is characterized by the ability to convert hydrogen peroxide to oxygen bubbles, and all of the soil isolates tested positive for the catalase enzyme ( Figure 1K ). We measured the degree of hemolytic activity by growing each isolate on blood agar ( Figure 1L ). We determined TEPI011 and TEPI014 to have alpha hemolytic activity characterized by a greenish discoloration, and TEPI012 to have beta hemolytic activity characterized by a clear zone around the patched isolate ( Figure 1L ).

Taken together, these results suggest that TEP011 is a Gram-negative, lactose-fermenting negative, catalase-positive, soil bacterium from the genus *Janthinobacterium* with alpha hemolytic activity. TEPI012 is a Gram-negative, lactose-fermenting negative, catalase-positive, soil bacteria from the genus *Pseudomonas* with beta hemolytic activity. TEP014 is a Gram-positive, catalase-positive, soil bacterium from the genus *Pseudoclavibacter* with alpha hemolytic activity. All three isolates exhibit antimicrobial activity against both Gram-positive and Gram-negative bacteria.

The secondary metabolites produced by soil bacteria have long been considered a treasure trove for antibiotic discovery (Clardy et al. 2009). Our findings that TEPI011 ( *Janthinobacterium* ) and TEPI012 ( *Pseudomonas* ) possess antimicrobial activity are consistent with previous studies identifying these soil bacteria to produce antibiotic compounds (Gionco et al. 2017; Inan&nbsp;Bektas et al. 2023; O’Sullivan et al. 1990). *Janthinobacterium* has a characteristic purple violet color due to the production of the secondary metabolite called violacein, which is also a potent antibiotic against both Gram-negative and Gram-positive bacteria (Choi et al. 2015; Inan&nbsp;Bektas et al. 2023). In addition, *Janthinobacterium* produces peptide lactone antibiotics called janthinocins, which have been shown to be effective bactericidal agents to treat pathogens such as methicillin-resistant *Staphylococcus aureus* (MRSA) (O’Sullivan et al. 1990). Likewise, *Pseudomonas* has been shown to produce several distinct antibiotic compounds including mupirocin, fluviols, and novel organometallic compounds (Gionco et al. 2017; Smirnov et al. 1997; Haas and Keel 2003). To our knowledge, *Pseudoclavibacter* has not previously been characterized as an antibiotic producer. Our biochemical characterization of *Pseudoclavibacter* is consistent with previous findings that *Pseudoclavibacter* is a Gram-positive, catalase-positive organism; however, the antimicrobial activity of *Pseudoclavibacter* reported in this study could potentially reveal a novel antibiotic compound (Oyaert et al. 2013; Pailhoriès et al. 2014).

&nbsp;

## Methods

*Soil Sample Collection*

Each soil sample was collected from locations on the Randolph-Macon College campus in Ashland, VA, near the root systems of various plants, at a soil depth of approximately 6 inches. The GPS location for each soil sample is as follows, TEPI011 37º 45' 43.2" N, 77º 28' 33.96 " W; TEPI012 37º 45' 44.64" N, 77º 28' 32.27" W; TEPI014 37º 45' 44" N, 77º 28' 33" W. Two tablespoons of each soil sample were collected in a sealable plastic bag and plated immediately.

*Cultivation of Soil Bacteria*

Each soil sample was diluted 1/10 by mixing 1 gram of soil with 9 mL of sterile water, then vortexed to mix. Four 10-fold serial dilutions were made in sterile water and 100 µL of each dilution (10 ^-1^ , 10 ^-2^ , 10 ^-3^ , 10 ^-4^ , 10 ^-5^ ) was plated onto either 10% Tryptic Soy Agar (TSA) or MacConkey agar. Plates were incubated at 22 °C for one week to produce single colonies of soil bacteria. A soil isolate library for each soil sample was created by picking sixteen individual colonies from serial dilution plates with a sterile toothpick and patching them onto a new 10% TSA plate in a 4 x 4 grid pattern. Soil isolate library plates were incubated at 22 °C for five days then stored at 4 °C.

*Antibiotic Screen*

The soil isolate library was screened for antibiotic-producing soil bacteria using the spread and patch technique (Handelsman et al., 2018). Two different tester strains, a Gram-negative tester strain ( *Escherichia coli,* Carolina Biological, #155065 *)* or Gram-positive tester strain ( *Bacillus subtilis* , Carolina Biological, # 154921) were first spread onto individual 10% TSA plates using a sterile swab. The soil isolate library for each sample was then patched in the same 4 x 4 grid pattern on top of the tester strain using a sterile toothpick. Plates were incubated at 22 °C for one week. Antibiotic-producing isolates were visually determined by selecting isolate patches with a clear ZOI where the growth of the tester strain had been inhibited. Three soil isolates (TEPI011, TEPI012, and TEP014) were selected for further study. The antibiotic screen was repeated by plating isolates individually in the center of a 60 mm 10% TSA plate spread with each tester strain ( *E.coli* and *B. subtilis* ) and incubating plates at 22 °C for one week to verify that each isolate could produce a ZOI when patched on top of each tester strain.

*Ethyl Acetate Chemical Extraction of Secondary Metabolites*

Each soil isolate was spread evenly across the entire surface of a 100 mm 10% TSA plate using a sterile swab and incubated at 22 °C for one week. Agar from each plate was then cut into approximately 1 cm ^2^ pieces using a sterile microspatula and collected in 250 mL glass Erlenmeyer flasks. To freeze-thaw cells, flasks were incubated at –80 °C for one week, then 15 mL of ethyl acetate and 10 mL of sterile water were added to frozen agar in the flask. Flasks were then covered with parafilm and aluminum foil and shaken at 200 rpm at 22 °C for one week. All the liquid from the flask was then transferred to a new 100 mL Erlenmeyer flask and left to sit for 3 minutes to allow for the liquid to separate into two layers: an aqueous bottom layer and an organic top layer composed of ethyl acetate and extracted secondary metabolites. If two layers were not visible, 1000 µL of sterile water were added to the liquid to distinguish the organic layer. The organic layer was carefully transferred to a scintillation vial and left uncapped for one week in a chemical fume hood to evaporate the ethyl acetate and leave only the extracted secondary metabolites in the vial. Each of the extracted secondary metabolites were reconstituted with 160 µL of methanol solvent.

*Disc Diffusion Assay*

To perform a disc diffusion assay with the extracted secondary metabolites, 30 µL of each reconstituted secondary metabolite mixture or methanol (negative control) was pipetted onto a sterile, ¼ inch diameter, blank paper disc (Carolina Biological Supply, 806491). Discs were allowed to dry for 10-15 minutes in a sterile biosafety cabinet. Tester strains ( *E.coli* or *B. subtilis* ) were spread on 60 mm 10% TSA agar plate using a sterile swab. Each treated disc was then placed in the center of the plate on top of the tester strain. Plates were incubated at 22 °C for one week and observed for development of a ZOI around the secondary metabolite treated discs. Two measurements of the ZOI diameter were taken though the center of the paper disc to calculate the mean ZOI.

*Gram Staining*

A dry mount slide of each soil isolate was prepared by spreading a single colony of bacteria in 5 µL drop of sterile water across a glass microscope slide. Slides were air-dried for 5 minutes then heat fixed. Gram stain (Carolina Biological Supply, 821050) was performed as follows: crystal violet (1 minute), iodine (1 minute), 95% ethanol (10 seconds), safranin (45 seconds). Stained sample was mounted with a coverslip and imaged at 100X magnification using a Nikon Eclipse E600 microscope equipped with a Leica MC 190 HD camera. Images were acquired with LASX 3.7.6 software.

*Biochemical tests (Catalase, Hemolysin, Lactose fermentation)*

Catalase test was performed on each isolate by spreading a colony on a glass microscope slide with a sterile pipette tip, then dropping 5 µL of hydrogen peroxide on top of the colony, then immediately observing for the formation of bubbles. Hemolysin assay was performed by patching each isolate with a sterile toothpick in the center of a 100 mm blood agar plate (VWR, 89405-024) and incubating the plate at 22 °C for one week. Lactose fermentation was determined by patching each isolate on a MacConkey agar plate (VWR, 89407-270) and incubating plate at 22 °C for one week.

*16S Ribosomal Gene Sequencing*

The genomic extraction and whole-genome sequencing using Oxford Nanopore Technology (ONT) Sequencing was outsourced to the company Eurofins Genomics. The genus of each isolate was determined using the open-source Basic Local Alignment Search Tool (BLAST) hosted by the National Center for Biotechnology Information (NCBI) (Altschul et al. 1990). Taxonomic identification was performed using BLASTn to compare the full-length 16S rRNA sequence to the NCBI 16S ribosomal RNA sequence (Bacteria and Archaea) database. Genus-level classification was assigned based on a established minimum sequence identity threshold of 95% (Janda and Abbott 2007). The 16S rRNA nucleotide sequences for all three soil isolates reported in this study are available in GeneBank under the following accession numbers: TEPI011 (PZ207879), TEPI012 (PZ207940), TEPI014 (PZ208169).

## Data Availability

Description: Disc diffusion assay for negative controls. Resource Type: Image. DOI: https://doi.org/10.22002/cgzwv-4zz19
